# Clinical outcomes and safety of zoledronic acid in castration-resistant metastatic prostate cancer with bone metastases: real-world data from Côte d’Ivoire

**DOI:** 10.3389/fonc.2026.1847737

**Published:** 2026-10-26

**Authors:** Bitti Addé Odo, Kouame Konan Yvon Kouassi, Pétiori Gningayou Laurence Toure, Gnakoury Alain Pacome Gnabro, Yenahaban Lazare Touré, Mohamed Kassir Agnide Madiou, Félix Mbarga Amougou, N'guessan Malan Prosper Mebiala, Fleur Audrey Sessegnon, Akissi Marie Barbara Yvonne Nogbou, Halourou Agrigna, Kalifa Sako, Moctar Toure, Innocent Adoubi

**Affiliations:** 1 Félix Houphouët-Boigny University, Faculty of Medicine, Abidjan, Côte d’Ivoire; 2 Centre Hospitalier Universitaire de Treichville, Abidjan, Côte d’Ivoire; 3 Alassane Ouattara University, Faculty of Medicine, Bouake, Côte d’Ivoire; 4 Centre Hospitalier Universitaire de Bouake, Bouake, Côte d’Ivoire

**Keywords:** bone metastases, mCRPC, resource-limited setting, skeletal-related events, zoledronic acid, Côte d’Ivoire

## Abstract

**Background:**

Zoledronic acid is used to prevent skeletal complications in patients with metastatic castration-resistant prostate cancer (mCRPC) and bone metastases. Real world data on its use in resource-limited setting, particularly in sub-saharan Africa, remain scarce.

**Aim:**

To describe clinical outcomes, skeletal-related events, safety and quality of life in patients with metastatic castration-resistant prostate cancer (mCRPC) and bone metastases treated with zoledronic acid in Côte d’Ivoire.

**Methods:**

We conducted a retrospective, single center cohort study including 144 patients with mCRPC and bone metastases treated with zoledronic acid at Treichville university Hospital between January 2019 and December 2021. The primary endpoint was skeletal event-free survival (SEFS) defined as the time from the first zoledronic acid administration to the first on-treatment skeletal related event (SRE). Patients without an SRE were censored at their last available follow-up.

**Results:**

The median age was 67 years (range: 42–91), and 133 patients (92.4%) were older than 50 years. Before zoledronic acid initiation, 59 patients (41.0%) had experienced at least one skeletal-related event. After treatment initiation, 30 patients (20.8%) experienced at least one new on-treatment SRE, whereas 114 (79.2%) did not. Among patients who experienced an SRE, the median time to the first event was 9 months (range 5-11). Median SEFS was not reached and the estimated 12-month SEFS was 79.2%. The most frequently documented adverse events were bone pain (18.8%), constipation (18.1%) and nausea (9.7%). FACT-G data at both baseline and 12-month were available for 129 patients (89.6%) and mean scores across all four FACT-G domains, as well as the total FACT-G score, were numerically lower at 12 months than at baseline.

**Conclusion:**

In this retrospective cohort of patients with mCRPC and bone metastases, 20.8% experienced a new SRE after zoledronic acid initiation, while median SEFS was not reached. FACT-G scores decreased during follow-up. These findings provide real-world data on zoledronic acid use in a resource-limited setting.

## Introduction

Prostate cancer is a major public health issue worldwide and in Côte d’Ivoire in particular (1). According to GLOBOCAN 2022, it ranked first among all cancers in Côte d’Ivoire, regardless of sex, with 4, 041 new cases and 2, 216 deaths reported in 2022 (1). In sub-Saharan Africa and in Côte d’Ivoire specifically, the disease is often diagnosed at an advanced stage (2). Prostate cancer is osteophilic, similar to other cancers such as breast and lung cancer (3). According to the literature, bone metastases are present in approximately 90% of patients with advanced prostate cancer (3, 4). The presence of bone metastases, combined with age-related bone demineralization or hormone therapy-induced bone loss, contributes to skeletal fragility (3). This fragility often results in skeletal-related events (SREs), including pathological fractures, spinal cord compression, and malignant hypercalcemia, all of which significantly increase disease-related morbidity and impair quality of life (3, 5, 6). Therefore, appropriate management of bone involvement is crucial in the therapeutic approach, and skeletal-related events must be closely monitored, promptly evaluated, and effectively treated (7). Although multiple treatment options are available to manage these complications, none are entirely satisfactory, even when used in combination (7). Consequently, patients with metastatic bone disease continue to pose a significant therapeutic challenge for clinicians. Several pharmacologic agents have been developed over the past two decades. Among them, denosumab (an anti-RANKL agent) and bisphosphonates (notably zoledronic acid) have become standard of care to reduce the incidence of skeletal-related events (8). Denosumab has demonstrated superiority over zoledronic acid in randomized controlled trials; however, it is not accessible in our setting (8). Zoledronic acid, which inhibits bone resorption, has an acceptable safety profile (7). It is readily available in our region and has been routinely used in clinical practice for more than five years. Despite its widespread use, no studies have yet assessed its clinical benefit in our local context. Therefore, we considered it necessary to conduct this study, with the overall objective of contributing to improved bone health in patients with metastatic prostate cancer involving the skeleton.

## Materials and methods

### Study design and setting

We conducted a retrospective, single-center cohort study with an analytical aim over a three-year period, from January 1, 2019, to December 31, 2021, in the Oncology Department of the University Hospital Center of Treichville. This department manages adult patients with solid tumors.

### Patient population

The study included all patients with histologically confirmed castration-resistant prostate adenocarcinoma treated with zoledronic acid.

### Inclusion and exclusion criteria

✓ Patients were eligible for inclusion if they met the following criteria:

  • Age over 18 years

  • Histologically confirmed prostate adenocarcinoma

  • Presence of at least one bone metastatic lesion

  • Evidence of castration resistance, defined as a disease progression despite maintenance of serum testosterone at castrate levels (<50 ng/dl or <1.7 nmol/l), with biochemical PSA progression and/or radiological progression.

  • Any performance status stage according to the WHO classification

  • Zoledronic acid treatment planned approximately every 28 days, with an allowable administration interval of 28–35 days.

  • Availability of clinical, biological and radiological follow-up data

✓ Patients with severe renal insufficiency (creatinine clearance <30 mL/min) were excluded.

### Sampling

We used an exhaustive sampling approach, including all eligible patients managed for metastatic castration-resistant prostate cancer with bone involvement during the study period who received zoledronic acid. A total of 144 patients met the eligibility criteria and constituted the final study cohort

### Treatment and method of administration

The administration of zoledronic acid was decided upon during a multidisciplinary team meeting as part of a comprehensive therapeutic management strategy. Zoledronic acid was administered at a dose of 4 mg/100 mL over 30 minutes, with an intended schedule of every 28 days and an allowable administration interval of 28–35 days.

Regarding systemic therapy, patients were receiving either docetaxel or abiraterone acetate at the time of zoledronic acid initiation. Docetaxel was the main systemic treatment option for patients eligible for chemotherapy and was continued until disease progression with dose adjustments according to clinical and biological tolerance. During the study period, access to newer androgen receptor pathway inhibitors was limited in our setting. Abiraterone acetate was available to a limited number of patients, whereas enzalutamide was not used. None of the patients received denosumab.

A dental or stomatological consultation was systematically performed before initiation of the treatment. Dental extractions were carried out when necessary to prevent the risk of jaw osteonecrosis.

### Data collection

A structured data collection form was developed, reviewed, and finalized. Patient data were collected from medical records (multidisciplinary team meetings, outpatient consultations, and hospitalizations). The information was recorded in a standardized, anonymized form containing all relevant study variables

### Handling of missing data

Analyses were performed using the available data for each variable. No imputation of missing data was performed

### Variables of interest

The studied parameters included:
  • Sociodemographic characteristics (age, personal and—when possible—family history of prostate, breast, or ovarian cancer, WHO performance status)
  • Disease characteristics at the time of castration resistance (mean PSA, metastatic sites, types of metastases, types of skeletal-related events before treatment). A single patient could present multiple types of bone lesions—sclerotic, lytic, or mixed—in varying proportions.
  • On-treatment skeletal outcomes (time to first on-treatment SRE, type of SRE)
  • Safety profile (adverse effects)
  • Follow-up and survival data (time to first on-treatment SRE, Vital status at last follow up, date of last follow-up and overall survival)
  • Quality-of-life assessed using the FACT-G questionnaire at baseline and at 12 months

### Study endpoints

The primary endpoint was skeletal event-free survival (SEFS), defined as the time from the first administration of zoledronic acid treatment to the occurrence of the first on-treatment skeletal-related event (SRE). SREs included pathological fracture, spinal cord compression, hypercalcemia, bone surgery or palliative radiotherapy to bone. Patients who did not experience an SRE during follow-up were censored at the date of their last available follow-up

Secondary endpoints included the occurrence and type of on treatment SREs, overall survival, adverse events and quality of life assessed using the functional Assessment of Cancer Therapy- General (FACT-G) questionnaire

### Operational definitions

The date of the first administration of zoledronic acid was considered the index date for the study.

Pretreatment skeletal-related events (SREs) were defined as SREs occurring before the first administration of zoledronic acid and were considered baseline characteristics.

On treatment SREs were defined as new SREs occurring after zoledronic acid initiation.

Palliative radiotherapy to bone was considered an SRE when it was delivered after zoledronic acid initiation for symptomatic bone metastases or skeletal complication requiring local symptom control. Radiotherapy delivered for other oncological indications was not classified as an SRE.

Overall survival (OS) was defined as the time from the first administration of zoledronic acid to death from any cause. Patients who were alive were censored at their last available follow-up

Safety assessment: Adverse events occurring during zoledronic acid treatment were graded according to the Common Terminology Criteria for Adverse Events (CTCAE), version 5.0. Individual patients could experience more than one type of adverse event and could therefore be counted in more than one category.

Quality of life assessment: quality of life was assessed using the Functional Assessment of Cancer Therapy- General (FACT-G) questionnaire at baseline and at 12 months. Higher scores indicated better quality of life. Longitudinal analyses were restricted to patients with evaluable data at both time points. Missing values were not imputed.

Hypercalcemia was defined as an albumin-corrected serum calcium level >105 mg/l.

### Data analysis

Data analysis was performed using IBM SPSS statistics, version 27.0.
  • Quantitative variables were described using mean and standard deviation or medians and ranges according to their distribution. Categorical variables were expressed as numbers and percentages.
  • Skeletal event-free survival (SEFS) and overall survival were estimated using the Kaplan–Meier method. For SEFS, time zero was the date of the first zoledronic acid administration, and the event was defined as the occurrence of the first on-treatment skeletal-related event (SRE). Patients who did not experience an SRE were censored at the date of their last available follow-up. For overall survival, time zero was also the first zoledronic acid administration. Death from any cause was considered the event and patients who were alive were censored at the date of their last available follow-up. Follow-up duration was calculated from the first administration of zoledronic acid to the last available follow-up or death. Patients were followed for a maximum of 24 months after zoledronic acid initiation
  • Factors associated with the occurrence of an on-treatment SRE were explored using univariate analyses. Categorical variables were compared using the Chi-square test or Fisher’s exact test when expected cell counts were small. Continuous variables were compared using student’s t-test.
  • Given the limited number of on-treatment SREs (n=30) relative to the number of potential explanatory variables, no multivariable Cox proportional hazards model was performed to avoid overfitting and unstable estimates.
  • For the longitudinal quality-of-life analysis, only patients with evaluable FACT-G data at both baseline and 12 months were included. FACT-G domain and total scores at baseline and 12 months were summarized descriptively using means, standard deviations and observed minimum and maximum values at each time point.
  • A p-value <0.05 was considered statistically significant.

### Ethical considerations

In accordance with the institutional procedures in place, authorization to conduct this retrospective study was granted by the medical and scientific Directorate of Treichville University Hospital in conjunction with the relevant administrative authorities of the institution, pending the establishment of an institutional ethics committee. No formal institutional ethics committee approval was obtained, and therefore no ethics committee approval number was available. Written informed consent for the use of clinical data for research purposes was obtained from the patients during their clinical care, with the data to be used after validation of the research protocol. Patient data confidentiality and respect for human dignity were maintained throughout data collection and processing

## Results

At the end of the study, 144 patients were included. The median age was 67 years (range: 42 to 91 years), with patients over the age of 50 accounting for 92.4% of the study population. A personal history of breast cancer was identified in six patients (4.2%), while a family history of breast and prostate cancer was noted in 6.9% and 11.1% of cases, respectively. According to the WHO performance status, the majority of patients had a score of 2 (54.9%), followed by a score of 3 (26.4%) (**Table 1**).

**Table 1:** Socio-demographic characteristics of the patients.

Parameter	n (%) or median (range)
Median age, years	67(42 - 91)
Age	
≤ 50 years	11(7.6)
> 50 years	133(92.4)
Personal history of breast cancer	6(4.2)
Family history of cancer	
Breast	10(6.9)
Ovary	1(0.7)
Prostate	16(11.1)
Breast and prostate	5(3.5)
Breast and ovary	1(0.7)
WHO Performance Status (PS)	
WHO PS 0	0(0.0)
WHO PS 1	22(15.3)
WHO PS 2	79(54.9)
WHO PS 3	38(26.4)
WHO PS 4	5(3.5)

PS, Performance Status; WHO, World Health Organization. The combined categories “breast and prostate” and “Breast and ovary” refer to patients with a family history of both cancer types. These combined categories were counted separately and were not included in the individual cancer categories. All family history categories were mutually exclusive.

Regarding disease characteristics at the time of castration resistance, the median PSA level was 280.5 ng/mL (range: 38 to 3200 ng/mL), and the median corrected serum calcium level was 102 mg/L (range: 86 to 145 mg/L). The median number of bone metastases was 6 lesions per patient (range: 3 to 8 lesions). The most frequent sites of bone metastases were spine (100% of cases), pelvis (93.1%), ribs (31.3%), and femur (24.3%). The predominant type of bone metastases was osteoblastic (sclerotic), observed in 88.9% of patients, and followed by osteolytic lesions in 76.4% of cases. Visceral metastases were present in 26 patients (18.1%) including brain metastases in 12 patients and lung metastases in 12 patients. Before zoledronic acid initiation, 59 patients (41.0%) had experienced at least one SRE. Based on the entire cohort (N = 144), the most frequent pretreatment SREs were hypercalcemia in 49 patients (34.0%), pathological fracture in 22 (15.3%) and spinal cord compression 20 (13.9%). Individual patients could experience more than one type of SRE (**Table 2**).

**Table 2:** Disease characteristics at the castration-resistant stage.

Parameter	n (%) or median (range)
Median PSA, ng/ml	280.5 (38- 3200)
Median corrected serum calcium, mg/l	102 (86 -145)
Median number of bone metastases	6 (3 - 8)
Bone metastatic sites	
Spine	144(100.0)
Pelvis	134(93.1)
Ribs	45(31.3)
Clavicle	29(20.1)
Femur	35(24.3)
Other sites (skull, humerus, tibia)	20(13.9)
Type of bone metastases	
Sclerotic	128(88.9)
Lytic	110(76.4)
Mixed	93(64.6)
Presence of visceral metastases	26(18.1)
Sites of associated visceral metastases	
Brain	12 (8.3)
Lung	12 (8.3)
Liver	2 (1.4)
Distant lymph nodes metastases	6 (4.2)
Skeletal-related events prior to zoledronic acid initiation	59(41.0)
Types of skeletal-related events prior to zoledronic acid initiation	
Pathological fracture	22(15.3)
Spinal cord compression	20(13.9)
Hypercalcemia	49(34.0)
Bone surgery	5(3.5)
Palliative radiotherapy to bone	10(6.9)
Systemic treatment	
Docetaxel	137(95.1)
Abiraterone acetate	7(4.9)

Percentages are calculated using the entire cohort (N = 144) as the denominator. Patients could experience more than one type of pretreatment SRE. Therefore these categories are not mutually exclusive.

After zoledronic acid initiation, 30 patients (20.8%) experienced at least one new on-treatment SRE, whereas 114 patients (79.2%) did not. Among patients who experienced an SRE, the median time to the first event was 9 months (range, 5 - 11). The most frequent on-treatment SREs were pathological fractures (9.0%) and spinal cord compression (8.3%). Regarding systemic treatment, 137 patients (95.1%) received docetaxel, whereas 7 patients (4.9%) received abiraterone acetate. None of the patients received denosumab (**Table 3**, **Figure 1**).

**Table 3:** On-treatment skeletal-related events after zoledronic acid initiation.

Parameter	n (%) or median (range)
Patients with ≥ 1 on-treatment SRE	30(20.8)
Patients without an on-treatment SRE	114 (79.2)
Median time to first on-treatment SRE among patients who experienced an SRE, months	9 (5-11)
Pathological fracture	13(9.0)
Spinal cord compression	12(8.3)
Palliative radiotherapy to bone	4(2.8)
Bone surgery	1(0.7)
Hypercalcemia	0(0.0)

**Figure 1:**
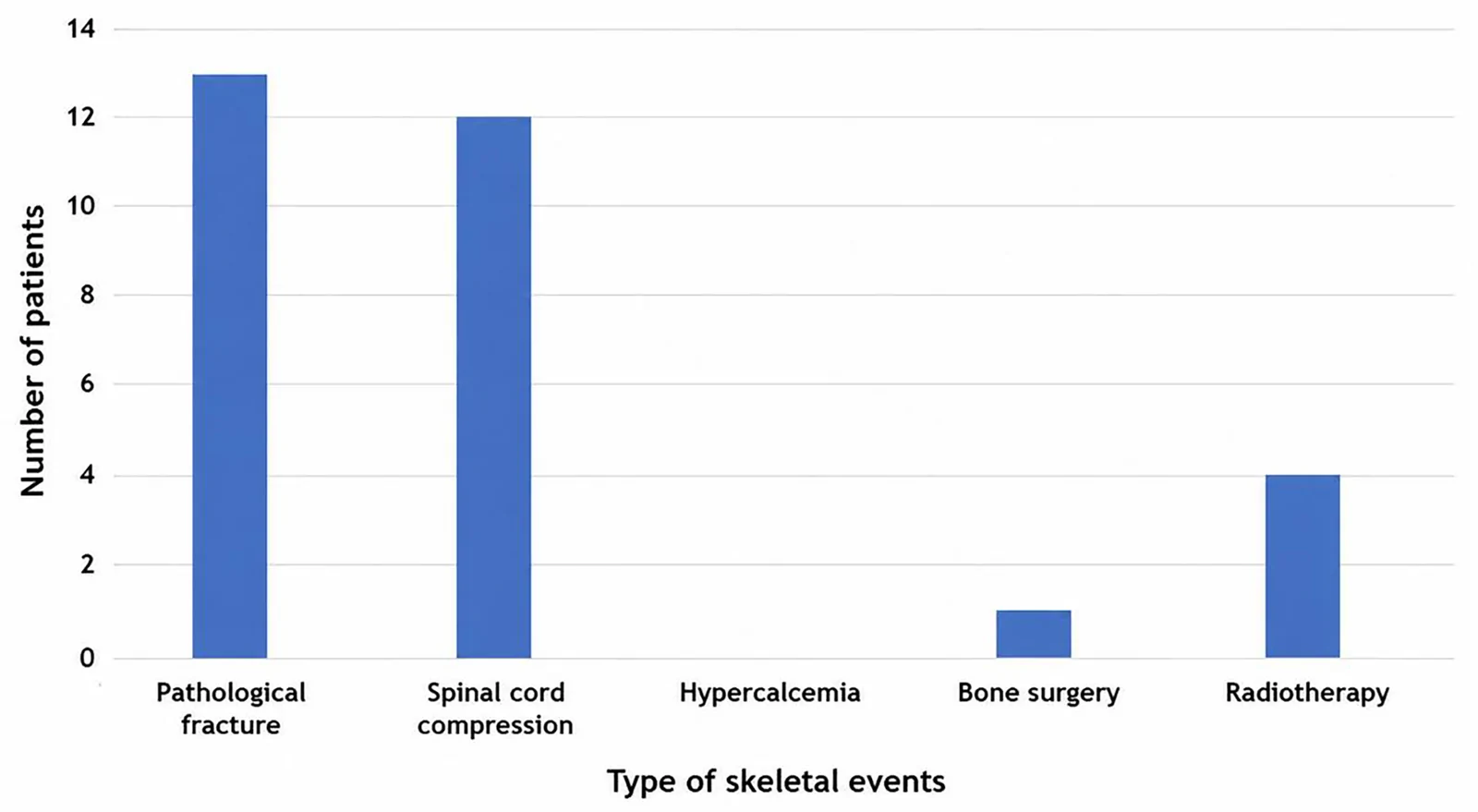
Types of skeletal-related events observed during zoledronic acid treatment.

Overall, 81 patients (56.3%) experienced at least one recorded adverse event during zoledronic acid treatment, with a total of 126 individual adverse events recorded. Of the 144 patients, 19 (13.2%) experienced at least one grade 3–4 adverse event. Individual patients could experience more than one type of adverse event. The most frequently reported adverse events were bone pain (18.8%), constipation (18.1%) and nausea (9.7%). Grade 3–4 adverse events mainly included bone pain (9.0%) and osteonecrosis of the jaw was observed in four patients (2.8%) (**Table 4**).

**Table 4:** Adverse events during zoledronic acid treatment (N = 144).

Toxicities	All grades, n (%)	Grade 3-4, n(%)
Osteonecrosis of the jaw	4 (2.8%)	4 (2.8%)
Nausea	14 (9.7%)	0(0.0%)
Vomiting	8 (5.6%)	3(2.1%)
Diarrhea	4 (2.8%)	2(1.4%)
Constipation	26 (18.1%)	0(0.0%)
Anorexia	4 (2.8%)	0(0.0%)
Fatigue	8 (5.6%)	0(0.0%)
Anemia	10 (6.9%)	2(1.4%)
Fever	11 (7.6%)	0(0.0%)
Dizziness	3 (2.1%)	0(0.0%)
Bone pain	27 (18.8%)	13(9.0%)
Kidney failure	7 (4.9%)	0(0.0%)

Individual patients could experience more than one adverse event and could therefore be counted in more than one category. Percentages were calculated using the entire cohort (N = 144) as a denominator.

The median follow-up duration, calculated directly from the first zoledronic acid administration to the last available follow-up or death, was 18 months (IQR, 15–22 months; range, 9–24 months). No patient without an on-treatment skeletal-related event (SRE) was censored before 12 months. Skeletal event-free survival was estimated in all 144 patients. Thirty patients experienced an event and 114 were censored. The Kaplan-Meier curve did not reach the 50% survival threshold. Therefore, median SEFS was not reached during follow-up. The estimated 12-month SEFS was 79.2%. Overall survival was estimated in all 144 patients. A total of 114 deaths were observed, while 30 patients were censored. Median overall survival from zoledronic acid initiation was 18 months (**Figures 2**, **3**).

**Figure 2:**
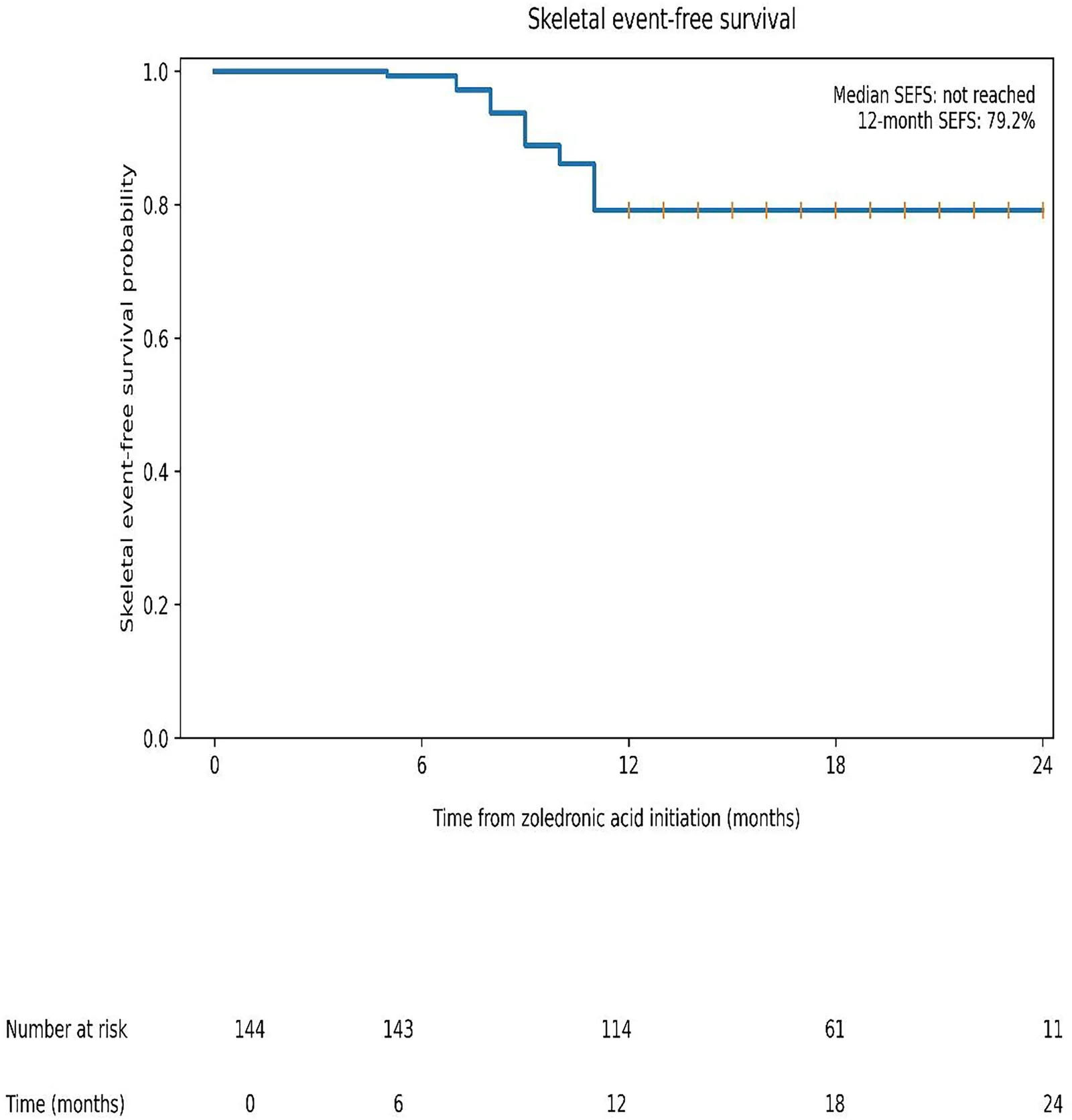
Kaplan–Meier estimate of skeletal event-free survival after zoledronic acid initiation. SEFS was defined as the time from the first zoledronic acid administration to the first on-treatment skeletal-related event. Patients without an SRE were censored at their last available follow-up. Median SEFS was not reached and the estimated 12-month SEFS was 79.2%. Tick marks indicate censored observations. Numbers at risk are shown below the curve.

**Figure 3:**
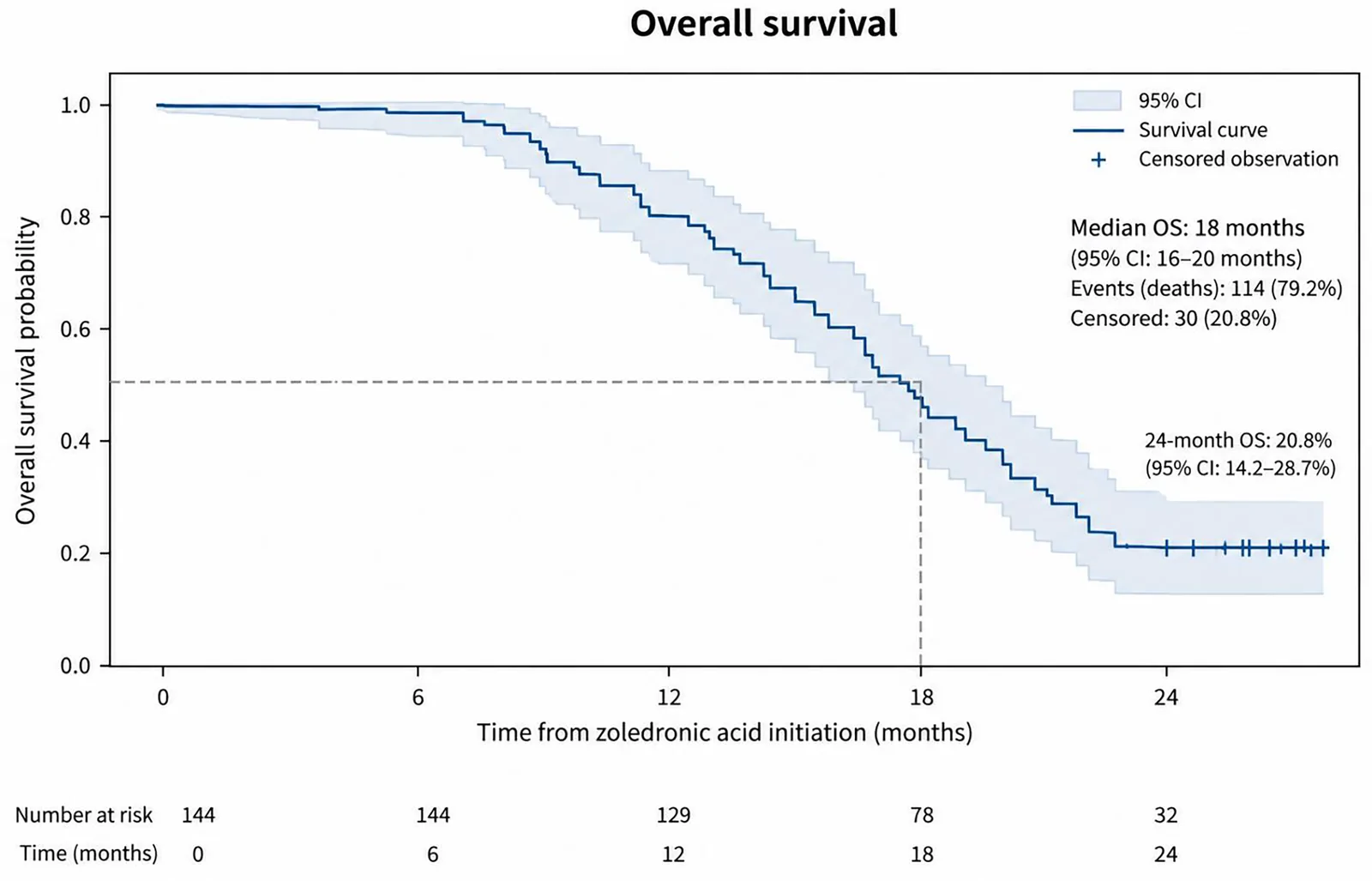
Kaplan–Meier curve of overall survival from zoledronic acid initiation. OS was defined as the time from the first administration of zoledronic acid to death from any cause. Patients who were alive were censored at their last available follow-up. Tick marks indicate censored observations. Numbers at risk are shown below the curve.

In univariate analysis, none of the factors evaluated was significantly associated with the occurrence of an on-treatment SRE. No significant association was observed according to bone lesion type: sclerotic (p=0.524), lytic (p=0.968) or mixed (p=0.486) (**Table 5**).

**Table 5:** Univariate analysis of factors associated with on-treatment skeletal-related events.

Parameters	SRE yes (n=30)	SRE no (n=114)	p-value
Age			0.459
≤ 50 years	1	10	
> 50 years	29	104	
WHO Performance Status			0.557
WHO PS 1	4	18	
WHO PS 2	14	65	
WHO PS 3	11	27	
WHO PS 4	1	4	
Initial PSA (Mean ± SD)	351.93 ± 266.80	402.71 ± 469.97	0.413
Type of bone metastases			
Sclerotic			0.524
Present	28	100	
absent	2	14	
Lytic			0.968
Present	23	87	
Absent	7	27	
Mixed			0.486
Present	21	72	
Absent	9	42	
Number of metastases (Mean ± SD)	5.58 ± 1.39	5.46 ± 1.24	0.779
Sites of metastases			
Pelvis			0.688
Present	29	105	
Absent	1	9	
Ribs			0.245
Present	12	33	
Absent	18	81	
Clavicle			0.983
Present	6	23	
Absent	24	91	
Femur			0.537
Present	6	29	
Absent	24	85	
Other sites (skull, humerus, tibia)			0.249
Present	2	18	
Absent	28	96	

Paired FACT-G data at both baseline and 12 months were available for 129 of 144 patients (89.6%). Follow-up FACT-G data at 12 months were unavailable or not evaluable for 15 (10.4%) patients. Mean scores across all four FACT-G domains, as well as the total FACT-G score, were numerically lower at 12 months than at baseline (**Table 6**).

**Table 6:** FACT-G quality of life domain scores at baseline and after 12 months of treatment (N = 129).

Parameters	Baseline: mean ± SD	Min-Max	12 months: mean ± SD	Min-max
FACT-G				
Physical well-being	21.96 ± 1.03	20-25	20.59 ± 0.797	19-23
Social/family well-being	22.18 ± 0.734	21-24	21.43 ± 0.496	21-22
Functional well-being	21.61 ± 1.388	18-25	20.76 ± 1.292	18-25
Emotional well-being	19.05 ± 1.211	17-21	18.04 ± 0.733	16-20
FACT-G total score	84.80 ± 2.560	80-94	80.81 ± 1.832	77-86

FACT-G, Functional Assessment of Cancer Therapy-General; SD, Standard deviation. The theoretical score ranges are 0–28 for physical, social/family and functional well-being; 0–24 for emotional well-being and 0–108 for the FACT-G total score. Higher scores indicate better quality of life. Analyses were based on paired assessments available at both baseline and 12 months (N = 129).

## Discussion

### Treatment outcomes

Skeletal complications represent a major concern in the management of patients with metastatic castration-resistant prostate cancer (mCRPC) and bone metastases (12). Zoledronic acid has demonstrated an ability to reduce skeletal complications in patients with metastatic castration-resistant prostate cancer and bone metastases in randomized studies (9, 13). In our study, the frequency of SREs observed during the follow-up appeared lower than that reported by Saad et al., who found a rate of 33.2% (9). However, this comparison should remain descriptive because of differences in study populations and concomitant systemic treatments.

Randomized studies have reported time-to-first-SRE outcomes in patients receiving bone-targeted agents (10, 13). However, these estimates cannot be directly compared with our skeletal event-free survival (SEFS), for which the median was not reached. Furthermore, the median time to the first SRE of 9 months reported in our study was calculated only among patients who experienced an event and does not represent the median SEFS of the entire cohort.

The nature of the complications observed in our cohort, predominantly pathological fractures and spinal cord compression, was consistent with the spectrum of SREs commonly described in mCRPC. Previous studies have also reported pathological fractures, radiotherapy to bone, bone surgery, hypercalcemia and spinal cord compression among the main manifestations of skeletal morbidity (8, 10, 14). The persistence of these events despite bone-targeted management highlights that such treatment is part of a broader therapeutic strategy and does not eliminate the need for careful clinical monitoring of skeletal complications.

Contemporary recommendations recognize bone-targeted agents as an important component of the management of patients with mCRPC and bone metastases who are at risk of skeletal complications. Zoledronic acid and denosumab are established options in this setting (8). In the randomized phase III trial directly comparing these two agents, denosumab delayed the occurrence of the first SRE compared with zoledronic acid, without demonstrating an improvement in overall survival (10). As none of the patients in our cohort received denosumab, our observations specifically reflect the use of zoledronic acid in our clinical setting.

The safety profile observed in our cohort was broadly consistent with previously reported data on zoledronic acid (8, 15, 16). Particular attention should nevertheless be paid to complications specifically associated with bisphosphonates, including renal toxicity and osteonecrosis of the jaw. The latter is a potentially serious complication that warrants preventive measures before and during treatment.

### Factors associated with the occurrence of skeletal-related events during treatment

None of the factors investigated was significantly associated with the occurrence of an SRE during treatment. However, the absence of a statistically significant association does not necessarily indicate the absence of predictors of skeletal complications in this population. It may partly reflect the limited statistical power resulting from the small number of events observed. Some studies have identified clinical or tumor-related factors potentially associated with the risk of skeletal complications, including age, clinical stage, number of bone lesions (11, 17). Differences in study populations may contribute to the discrepancies between these findings and ours.

The limited number of SREs observed during follow-up also represented an important constraint for multivariable analysis. In this context, simultaneously introducing several variables into a Cox model would have increased the risk of overfitting and unstable estimates.

### Survival

The median overall survival in our cohort was 18 months. In the randomized trial by Fizazi et al., median overall survival was 19.8 months in the zoledronic acid group (10). However, direct comparisons should be interpreted cautiously because of differences in patient populations and concomitant treatments.

### Quality of life

The decrease in FACT-G scores observed during follow-up involved all assessed domains and the total score. However, these findings should be interpreted cautiously. The clinical relevance of the observed changes remains uncertain, as the minimal clinically important difference was not assessed in our study.

Furthermore, quality of life in patients with mCRPC is likely influenced by multiple factors. Disease progression, skeletal metastatic burden, the occurrence of skeletal complications and adverse effects of concomitant oncologic treatments may all affect the different FACT-G domains. In the absence of a comparator group, the decrease in scores observed during follow-up cannot therefore be attributed to zoledronic acid. Rather, these findings should be considered as describing the longitudinal evolution of quality of life in a population with advanced metastatic disease.

In sub-Saharan Africa, bone metastases are frequently observed in patients with advanced prostate cancer, while data on the prevention and management of skeletal-related events remain limited (18). Zoledronic acid is one of the available options for SRE prevention in settings where access to different bone-targeted agents may be limited.

### Study limitations

This study has several limitations.

First, its retrospective, single-center design and the absence of a comparator group limit the ability to establish a causal relationship between zoledronic acid administration and the observed clinical outcomes.

Second, concomitant oncologic treatments were not uniform, particularly because access to newer androgen receptor pathway inhibitors such as abiraterone and enzalutamide was limited in our setting. In addition, the precise time of docetaxel initiation relative to zoledronic acid was not available from the retrospective data. Therefore, the respective influence of systemic therapy and bone-targeted treatment on SRE occurrence could not be assessed. Differences in systemic treatment may also have influenced survival independently of bone-target therapy.

Third, the relatively small number of on-treatment SREs limited the statistical power of the analyses exploring factors associated with SRE occurrence and precluded the use of a reliable multivariable model.

The longitudinal quality of life analysis was restricted to patients with FACT-G assessments available at both time points and did not include an assessment of the clinical relevance of the observed changes. Finally, the retrospective collection of adverse events from medical records carries a risk of underreporting some toxicities.

Despite these limitations, this study provides real-world data on the use of zoledronic acid in patients with metastatic castration-resistant prostate cancer and bone metastases in a resource-limited setting, a population for which data from routine clinical practice remain limited.

## Conclusion

This study provides real-world data on the use of zoledronic acid in patients with mCRPC and bone metastases in a resource-limited setting. Zoledronic acid remains a treatment option used for the prevention of skeletal complications where access to other bone-targeted agents is limited. Larger prospective studies are needed to confirm these findings.

## Data Availability

The raw data supporting the conclusions of this article will be made available by the authors, without undue reservation.
